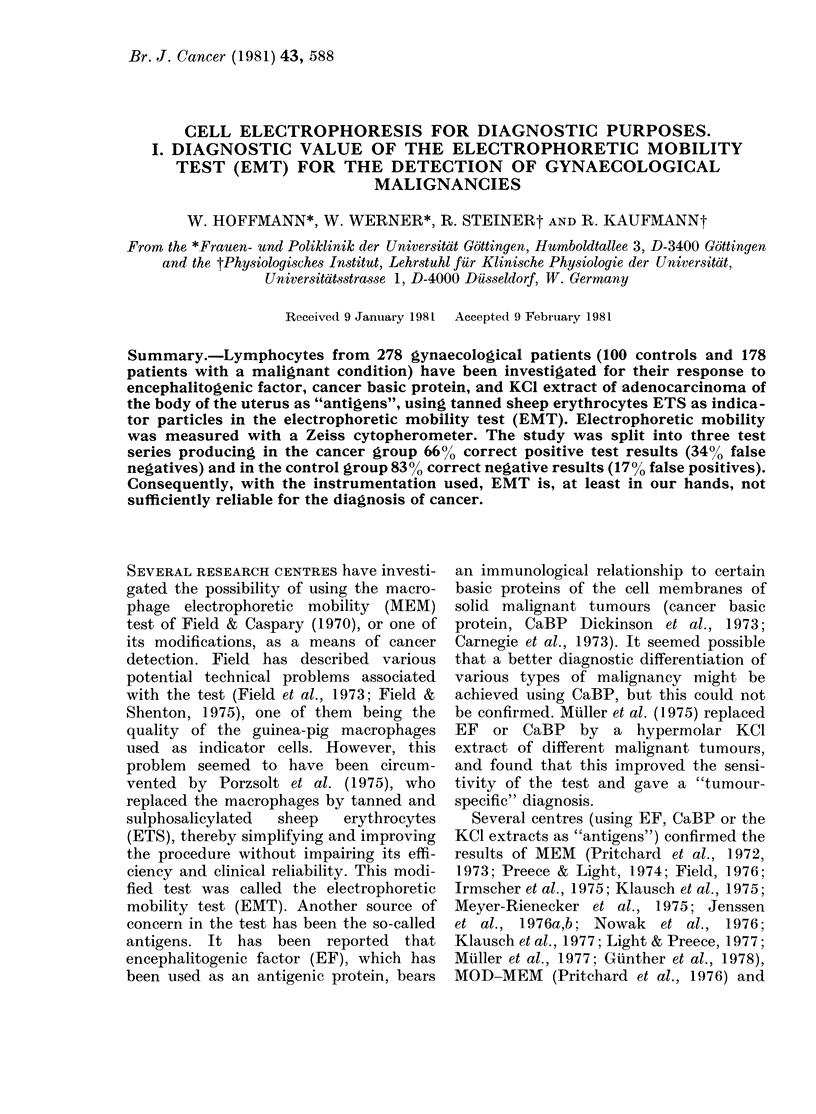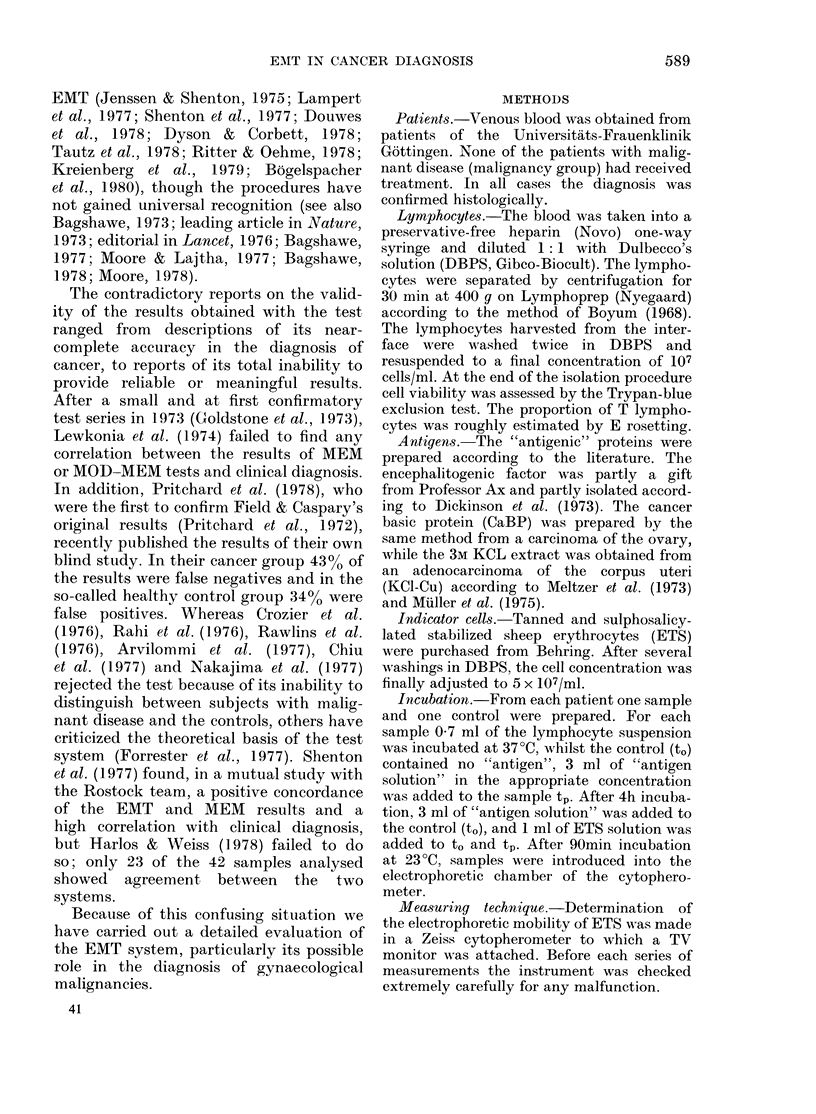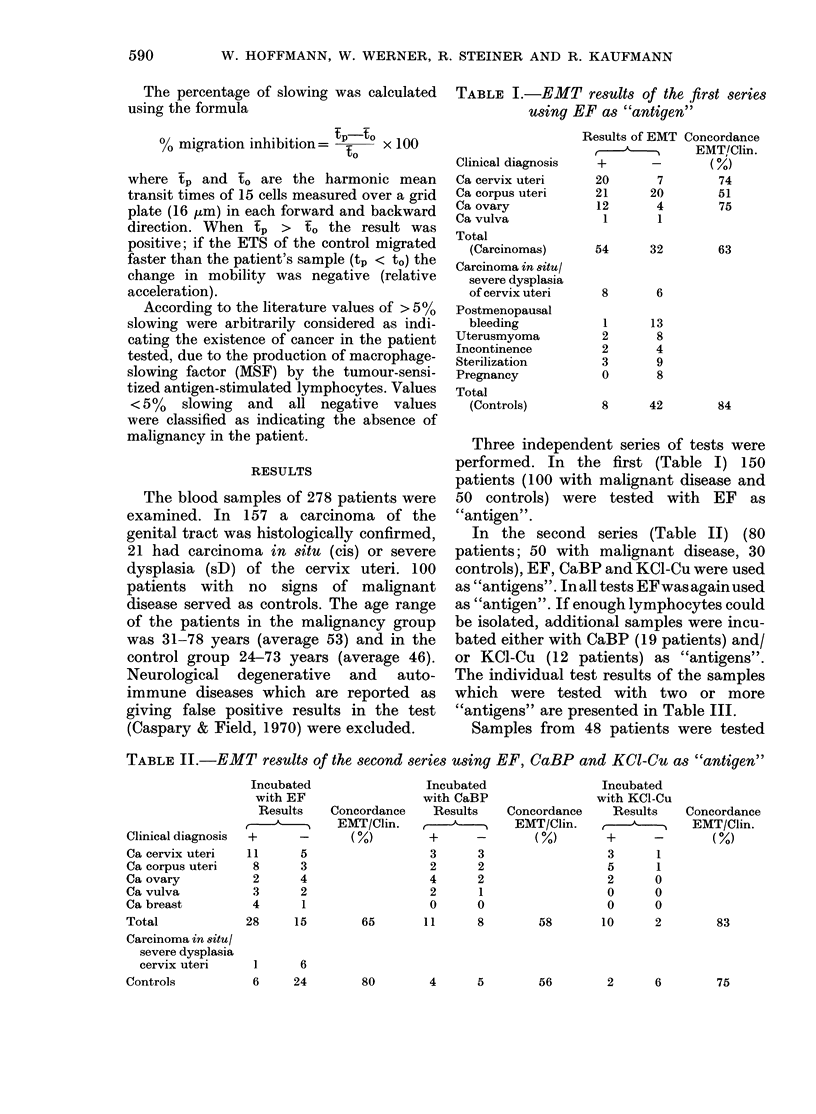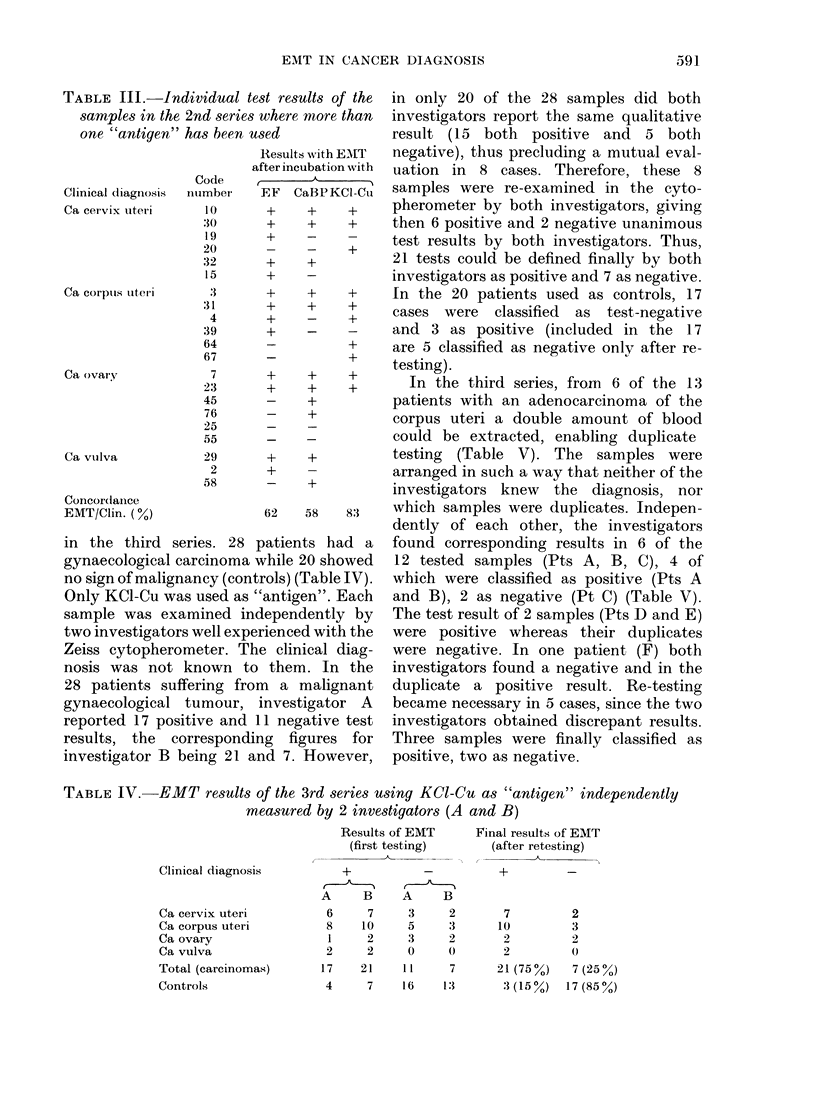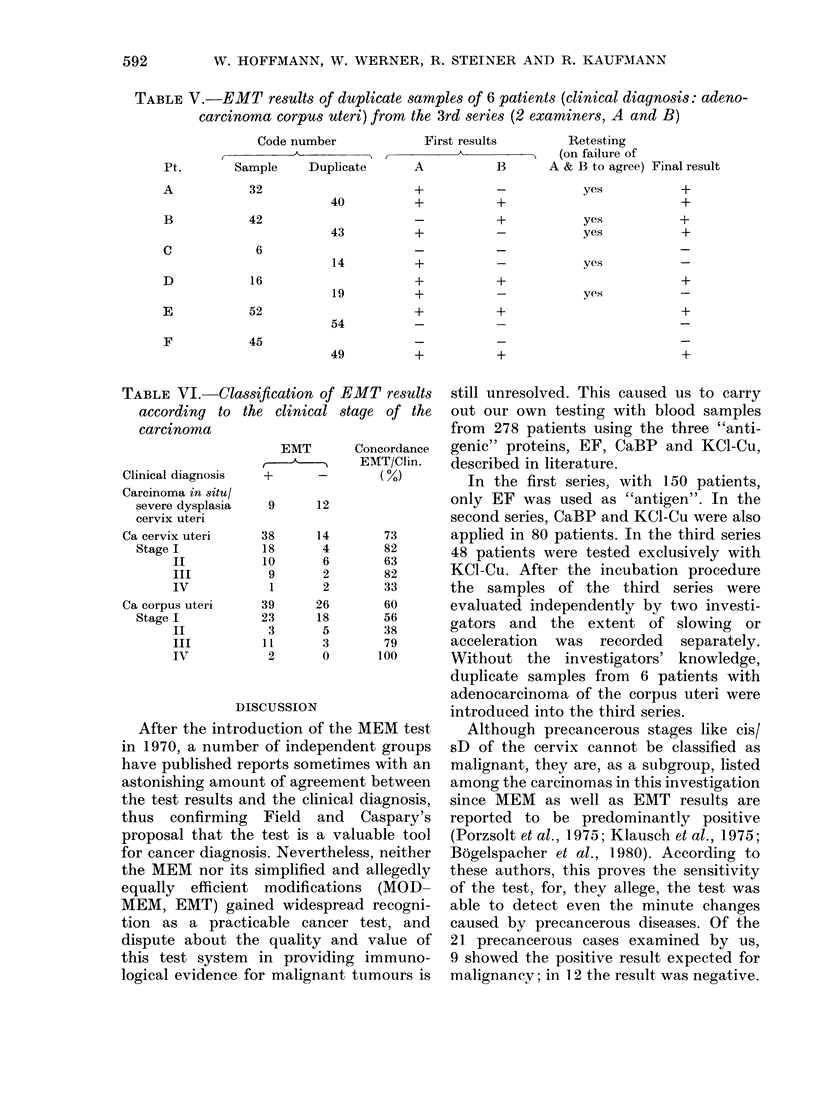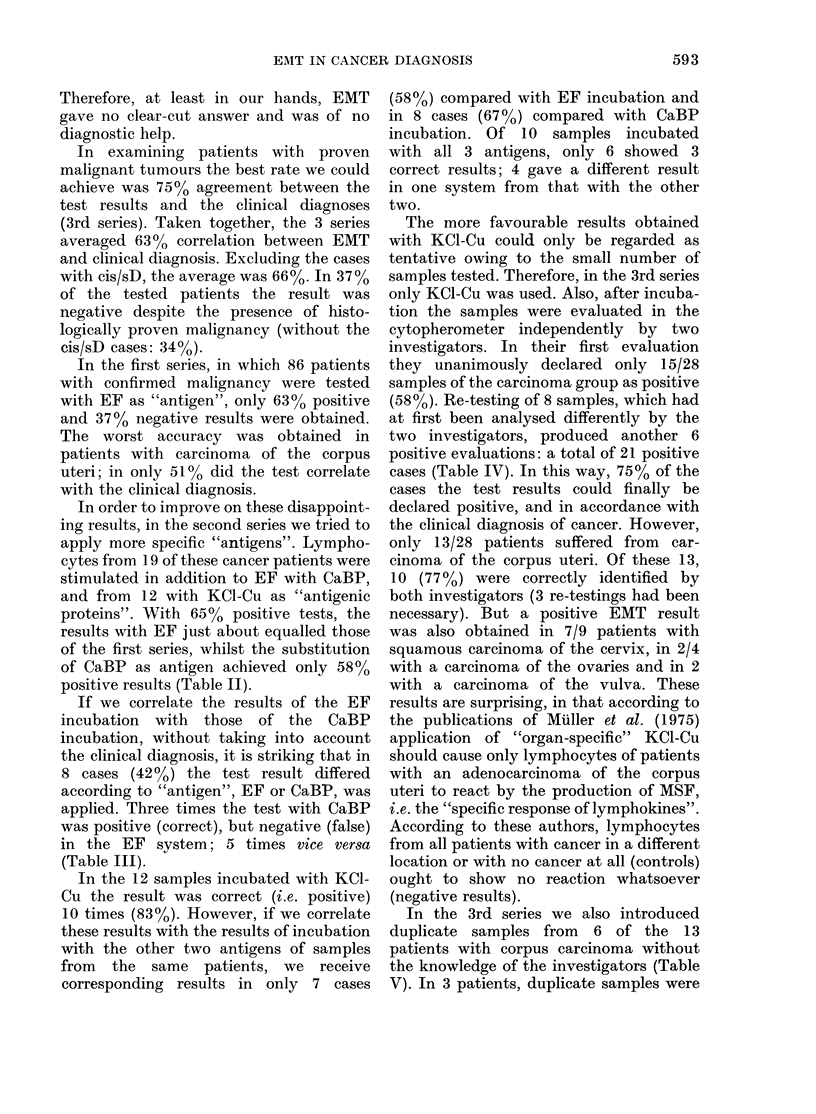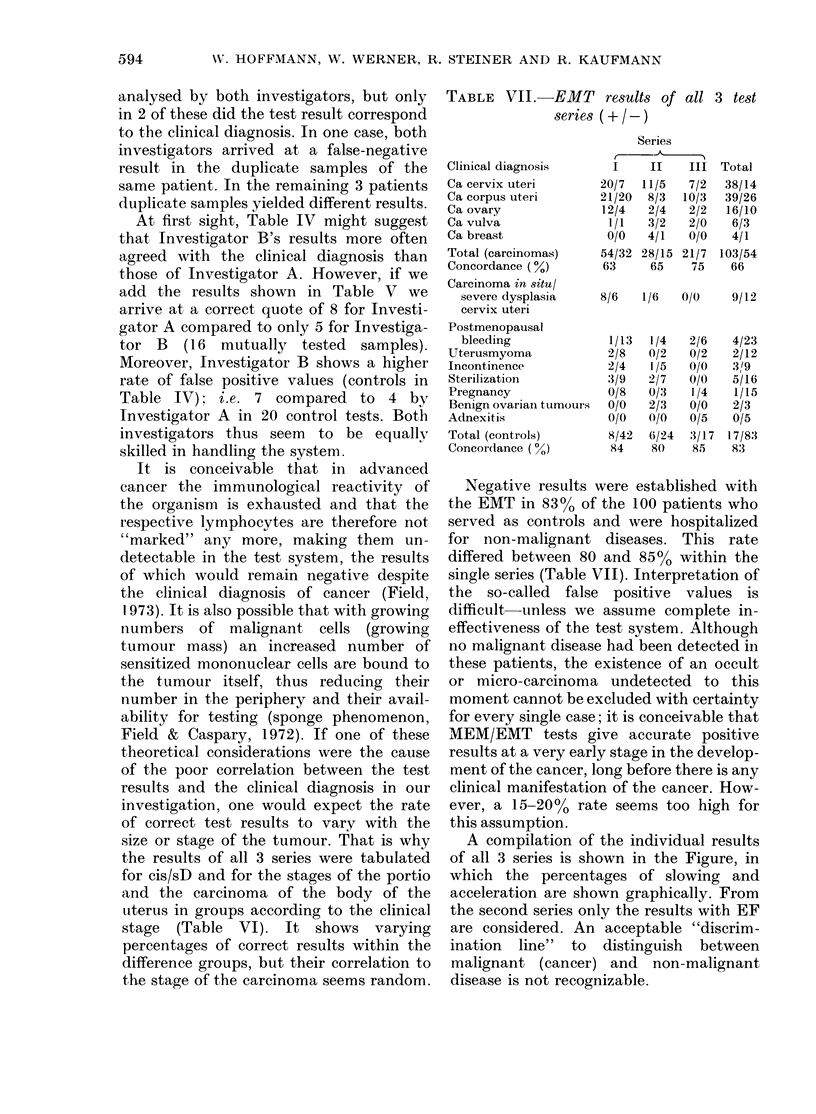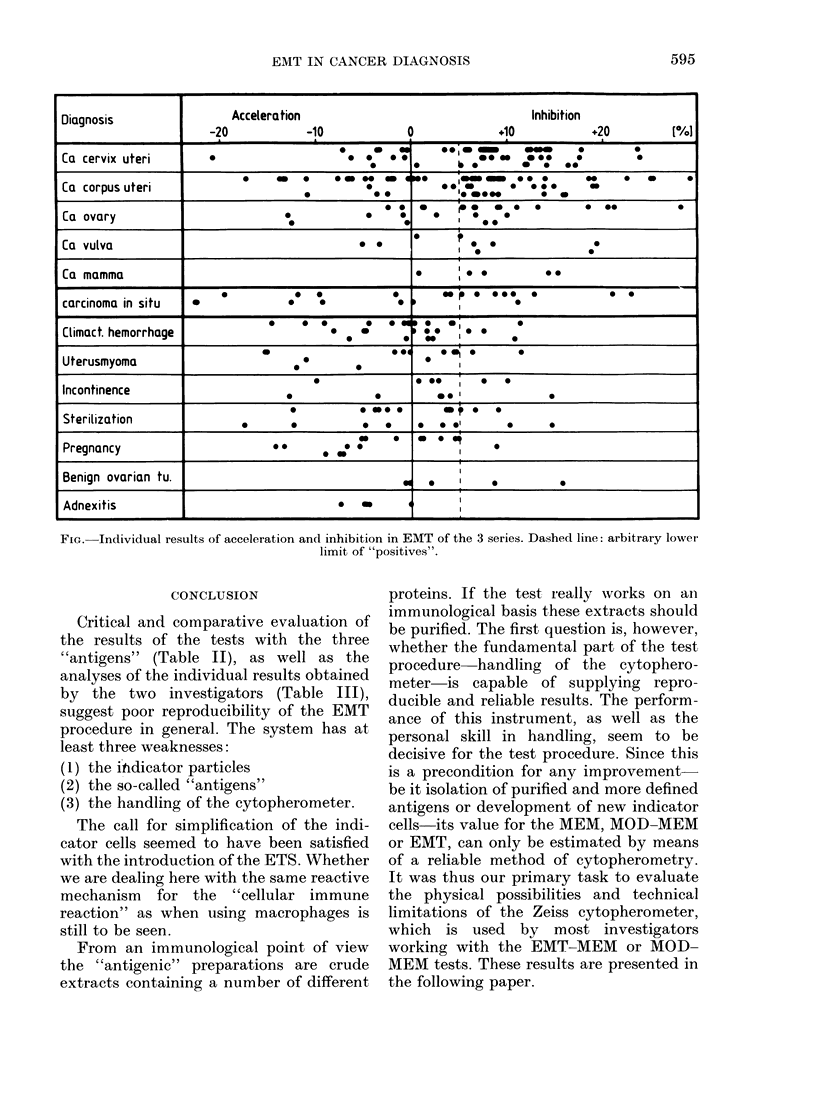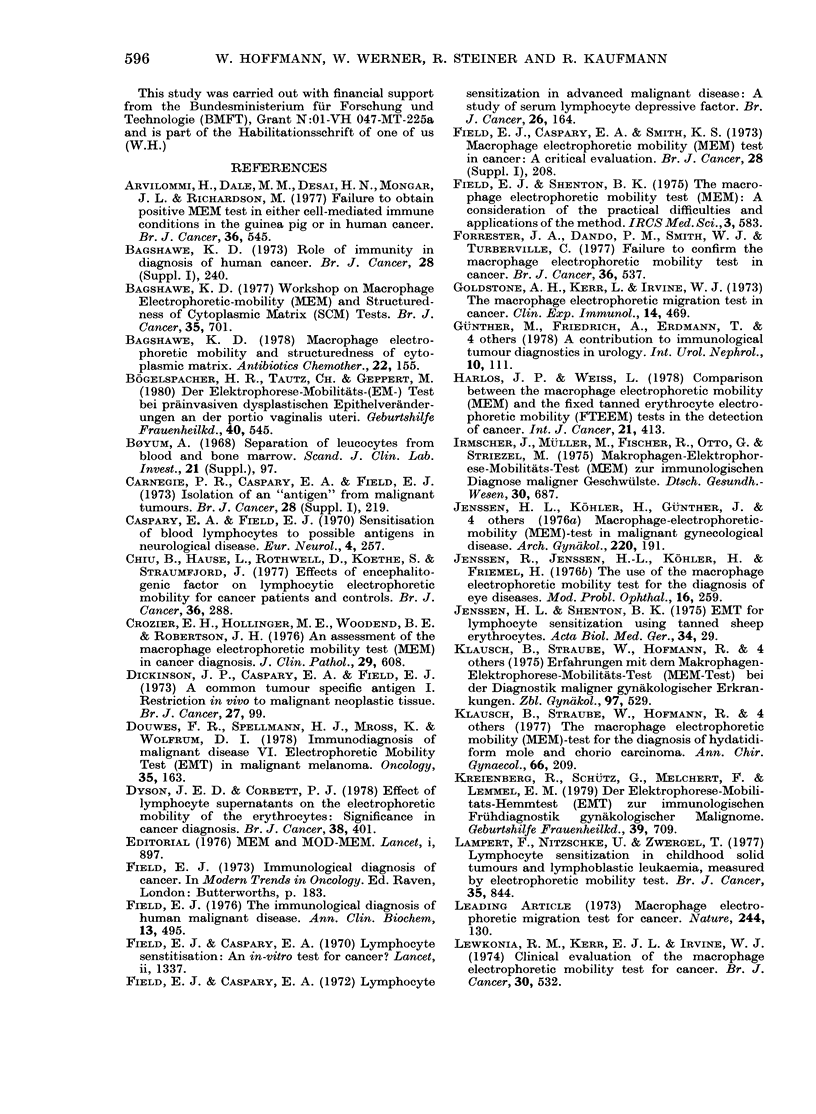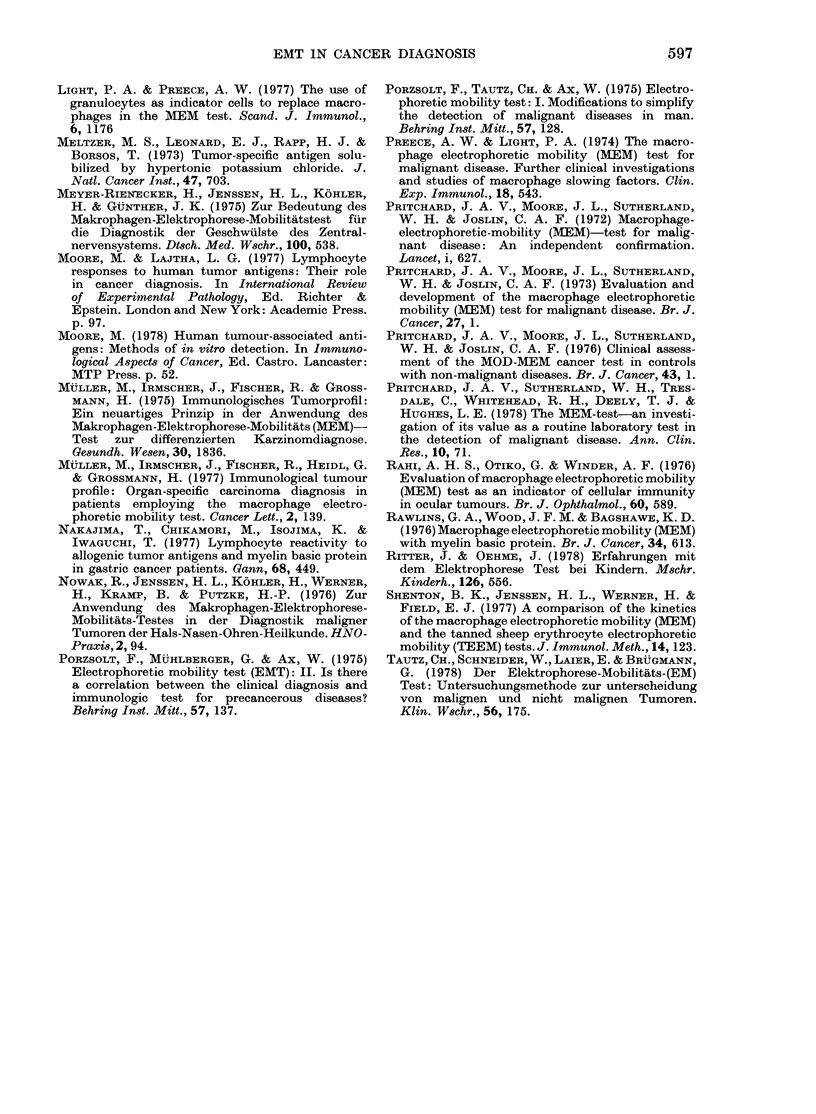# Cell electrophoresis for diagnostic purposes. I. Diagnostic value of the electrophoretic mobility test (EMT) for the detection of gynaecological malignancies.

**DOI:** 10.1038/bjc.1981.88

**Published:** 1981-05

**Authors:** W. Hoffmann, W. Werner, R. Steiner, R. Kaufmann

## Abstract

Lymphocytes from 278 gynaecological patients (100 controls and 178 patients with a malignant condition) have been investigated for their response to encephalitogenic factor, cancer basic protein, and KCl extract of adenocarcinoma of the body of the uterus as "antigens", using tanned sheep erythrocytes ETS as indicator particles in the electrophoretic mobility test (EMT). Electrophoretic mobility was measured with a Zeiss cytopherometer. The study was split into three test series producing in the cancer group 66% correct positive test results (34% false negatives) and in the control group 83% correct negative results (17% false positives). Consequently, with the instrumentation used, EMT is, at least in our hands, not sufficiently reliable for the diagnosis of cancer.


*Br. J. Cancer* (1981) **43**, 588

### CELL ELECTROPHORESIS FOR DIAGNOSTIC PURPOSES. I. DIAGNOSTIC VALUE OF THE ELECTROPHORETIC MOBILITY TEST (EMT) FOR THE DETECTION OF GYNAECOLOGICAL MALIGNANCIES

W. HOFFMANN*, W. WERNER*, R. STEINER† AND R. KAUFMANN†

*From the *Frauen- und Poliklinik der Universität Göttingen, Humboldtallee 3, D-3400 Göttingen and the †Physiologisches Institut, Lehrstuhl für Klinische Physiologie der Universität, Universitätsstrasse 1, D-4000 Düsseldorf, W. Germany*

Received 9 January 1981 Accepted 9 February 1981

**Summary.—Lymphocytes from 278 gynaecological patients (100 controls and 178 patients with a malignant condition) have been investigated for their response to encephalitogenic factor, cancer basic protein, and KCl extract of adenocarcinoma of the body of the uterus as "antigens", using tanned sheep erythrocytes ETS as indicator particles in the electrophoretic mobility test (EMT). Electrophoretic mobility was measured with a Zeiss cytopherometer. The study was split into three test series producing in the cancer group 66% correct positive test results (34% false negatives) and in the control group 83% correct negative results (17% false positives). Consequently, with the instrumentation used, EMT is, at least in our hands, not sufficiently reliable for the diagnosis of cancer.**

SEVERAL RESEARCH CENTRES have investigated the possibility of using the macrophage electrophoretic mobility (MEM) test of Field & Caspary (1970), or one of its modifications, as a means of cancer detection. Field has described various potential technical problems associated with the test (Field *et al.*, 1973; Field & Shenton, 1975), one of them being the quality of the guinea-pig macrophages used as indicator cells. However, this problem seemed to have been circumvented by Porzsolt *et al.* (1975), who replaced the macrophages by tanned and sulphosalicylated sheep erythrocytes (ETS), thereby simplifying and improving the procedure without impairing its efficiency and clinical reliability. This modified test was called the electrophoretic mobility test (EMT). Another source of concern in the test has been the so-called antigens. It has been reported that encephalitogenic factor (EF), which has been used as an antigenic protein, bears an immunological relationship to certain basic proteins of the cell membranes of solid malignant tumours (cancer basic protein, CaBP Dickinson *et al.*, 1973; Carnegie *et al.*, 1973). It seemed possible that a better diagnostic differentiation of various types of malignancy might be achieved using CaBP, but this could not be confirmed. Müller *et al.* (1975) replaced EF or CaBP by a hypermolar KCl extract of different malignant tumours, and found that this improved the sensitivity of the test and gave a "tumour-specific" diagnosis.

Several centres (using EF, CaBP or the KCl extracts as "antigens") confirmed the results of MEM (Pritchard *et al.*, 1972, 1973; Preece & Light, 1974; Field, 1976; Irmscher *et al.*, 1975; Klausch *et al.*, 1975; Meyer-Rienecker *et al.*, 1975; Jenssen *et al.*, 1976*a,b*; Nowak *et al.*, 1976; Klausch *et al.*, 1977; Light & Preece, 1977; Müller *et al.*, 1977; Günther *et al.*, 1978), MOD-MEM (Pritchard *et al.*, 1976) and

EMT (Jenssen & Shenton, 1975; Lampert *et al.*, 1977; Shenton *et al.*, 1977; Douwes *et al.*, 1978; Dyson & Corbett, 1978; Tautz *et al.*, 1978; Ritter & Oehme, 1978; Kreienberg *et al.*, 1979; Bögelspacher *et al.*, 1980), though the procedures have not gained universal recognition (see also Bagshawe, 1973; leading article in *Nature*, 1973; editorial in *Lancet*, 1976; Bagshawe, 1977; Moore & Lajtha, 1977; Bagshawe, 1978; Moore, 1978).

The contradictory reports on the validity of the results obtained with the test ranged from descriptions of its near-complete accuracy in the diagnosis of cancer, to reports of its total inability to provide reliable or meaningful results. After a small and at first confirmatory test series in 1973 (Goldstone *et al.*, 1973), Lewkonia *et al.* (1974) failed to find any correlation between the results of MEM or MOD–MEM tests and clinical diagnosis. In addition, Pritchard *et al.* (1978), who were the first to confirm Field & Caspary's original results (Pritchard *et al.*, 1972), recently published the results of their own blind study. In their cancer group 43% of the results were false negatives and in the so-called healthy control group 34% were false positives. Whereas Crozier *et al.* (1976), Rahi *et al.* (1976), Rawlins *et al.* (1976), Arvilommi *et al.* (1977), Chiu *et al.* (1977) and Nakajima *et al.* (1977) rejected the test because of its inability to distinguish between subjects with malignant disease and the controls, others have criticized the theoretical basis of the test system (Forrester *et al.*, 1977). Shenton *et al.* (1977) found, in a mutual study with the Rostock team, a positive concordance of the EMT and MEM results and a high correlation with clinical diagnosis, but Harlos & Weiss (1978) failed to do so; only 23 of the 42 samples analysed showed agreement between the two systems.

Because of this confusing situation we have carried out a detailed evaluation of the EMT system, particularly its possible role in the diagnosis of gynaecological malignancies.

### METHODS

*Patients.*—Venous blood was obtained from patients of the Universitäts-Frauenklinik Göttingen. None of the patients with malignant disease (malignancy group) had received treatment. In all cases the diagnosis was confirmed histologically.

*Lymphocytes.*—The blood was taken into a preservative-free heparin (Novo) one-way syringe and diluted 1 : 1 with Dulbecco's solution (DBPS, Gibco-Biocult). The lymphocytes were separated by centrifugation for 30 min at 400 *g* on Lymphoprep (Nyegaard) according to the method of Boyum (1968). The lymphocytes harvested from the interface were washed twice in DBPS and resuspended to a final concentration of $10^7$ cells/ml. At the end of the isolation procedure cell viability was assessed by the Trypan-blue exclusion test. The proportion of T lymphocytes was roughly estimated by E rosetting.

*Antigens.*—The "antigenic" proteins were prepared according to the literature. The encephalitogenic factor was partly a gift from Professor Ax and partly isolated according to Dickinson *et al.* (1973). The cancer basic protein (CaBP) was prepared by the same method from a carcinoma of the ovary, while the 3M KCL extract was obtained from an adenocarcinoma of the corpus uteri (KCl-Cu) according to Meltzer *et al.* (1973) and Müller *et al.* (1975).

*Indicator cells.*—Tanned and sulphosalicylated stabilized sheep erythrocytes (ETS) were purchased from Behring. After several washings in DBPS, the cell concentration was finally adjusted to $5 \times 10^7$/ml.

*Incubation.*—From each patient one sample and one control were prepared. For each sample 0·7 ml of the lymphocyte suspension was incubated at 37 °C, whilst the control ($t_0$) contained no "antigen", 3 ml of "antigen solution" in the appropriate concentration was added to the sample $t_p$. After 4h incubation, 3 ml of "antigen solution" was added to the control ($t_0$), and 1 ml of ETS solution was added to $t_0$ and $t_p$. After 90min incubation at 23 °C, samples were introduced into the electrophoretic chamber of the cytopherometer.

*Measuring technique.*—Determination of the electrophoretic mobility of ETS was made in a Zeiss cytopherometer to which a TV monitor was attached. Before each series of measurements the instrument was checked extremely carefully for any malfunction.

The percentage of slowing was calculated using the formula

$$\% \text{ migration inhibition} = \frac{\bar{t}_p - \bar{t}_o}{\bar{t}_o} \times 100$$

where $\bar{t}_p$ and $\bar{t}_o$ are the harmonic mean transit times of 15 cells measured over a grid plate (16 $\mu$m) in each forward and backward direction. When $\bar{t}_p > \bar{t}_o$ the result was positive; if the ETS of the control migrated faster than the patient's sample ($t_p < t_o$) the change in mobility was negative (relative acceleration).

According to the literature values of $>5\%$ slowing were arbitrarily considered as indicating the existence of cancer in the patient tested, due to the production of macrophage-slowing factor (MSF) by the tumour-sensitized antigen-stimulated lymphocytes. Values $<5\%$ slowing and all negative values were classified as indicating the absence of malignancy in the patient.

### RESULTS

The blood samples of 278 patients were examined. In 157 a carcinoma of the genital tract was histologically confirmed, 21 had carcinoma *in situ* (cis) or severe dysplasia (sD) of the cervix uteri. 100 patients with no signs of malignant disease served as controls. The age range of the patients in the malignancy group was 31–78 years (average 53) and in the control group 24–73 years (average 46). Neurological degenerative and autoimmune diseases which are reported as giving false positive results in the test (Caspary & Field, 1970) were excluded.

TABLE I.—*EMT results of the first series using EF as "antigen"*

| Clinical diagnosis | Results of EMT + | Results of EMT − | Concordance EMT/Clin. (%) |
|---|---|---|---|
| Ca cervix uteri | 20 | 7 | 74 |
| Ca corpus uteri | 21 | 20 | 51 |
| Ca ovary | 12 | 4 | 75 |
| Ca vulva | 1 | 1 | |
| Total (Carcinomas) | 54 | 32 | 63 |
| Carcinoma *in situ*/ severe dysplasia of cervix uteri | 8 | 6 | |
| Postmenopausal bleeding | 1 | 13 | |
| Uterusmyoma | 2 | 8 | |
| Incontinence | 2 | 4 | |
| Sterilization | 3 | 9 | |
| Pregnancy | 0 | 8 | |
| Total (Controls) | 8 | 42 | 84 |

Three independent series of tests were performed. In the first (Table I) 150 patients (100 with malignant disease and 50 controls) were tested with EF as "antigen".

In the second series (Table II) (80 patients; 50 with malignant disease, 30 controls), EF, CaBP and KCl-Cu were used as "antigens". In all tests EF was again used as "antigen". If enough lymphocytes could be isolated, additional samples were incubated either with CaBP (19 patients) and/or KCl-Cu (12 patients) as "antigens". The individual test results of the samples which were tested with two or more "antigens" are presented in Table III.

Samples from 48 patients were tested

TABLE II.—*EMT results of the second series using EF, CaBP and KCl-Cu as "antigen"*

| Clinical diagnosis | Incubated with EF Results + | Incubated with EF Results − | Concordance EMT/Clin. (%) | Incubated with CaBP Results + | Incubated with CaBP Results − | Concordance EMT/Clin. (%) | Incubated with KCl-Cu Results + | Incubated with KCl-Cu Results − | Concordance EMT/Clin. (%) |
|---|---|---|---|---|---|---|---|---|---|
| Ca cervix uteri | 11 | 5 | | 3 | 3 | | 3 | 1 | |
| Ca corpus uteri | 8 | 3 | | 2 | 2 | | 5 | 1 | |
| Ca ovary | 2 | 4 | | 4 | 2 | | 2 | 0 | |
| Ca vulva | 3 | 2 | | 2 | 1 | | 0 | 0 | |
| Ca breast | 4 | 1 | | 0 | 0 | | 0 | 0 | |
| Total | 28 | 15 | 65 | 11 | 8 | 58 | 10 | 2 | 83 |
| Carcinoma *in situ*/ severe dysplasia cervix uteri | 1 | 6 | | | | | | | |
| Controls | 6 | 24 | 80 | 4 | 5 | 56 | 2 | 6 | 75 |

TABLE III.—*Individual test results of the samples in the 2nd series where more than one "antigen" has been used*

| Clinical diagnosis | Code number | Results with EMT after incubation with: EF | CaBP | KCl-Cu |
|---|---|---|---|---|
| Ca cervix uteri | 10 | + | + | + |
| | 30 | + | + | + |
| | 19 | + | − | − |
| | 20 | − | − | + |
| | 32 | + | + | |
| | 15 | + | − | |
| Ca corpus uteri | 3 | + | + | + |
| | 31 | + | + | + |
| | 4 | + | − | + |
| | 39 | + | − | − |
| | 64 | − | | + |
| | 67 | − | | + |
| Ca ovary | 7 | + | + | + |
| | 23 | + | + | + |
| | 45 | − | + | |
| | 76 | − | + | |
| | 25 | − | − | |
| | 55 | − | − | |
| Ca vulva | 29 | + | + | |
| | 2 | + | − | |
| | 58 | − | + | |
| Concordance EMT/Clin. (%) | | 62 | 58 | 83 |

in the third series. 28 patients had a gynaecological carcinoma while 20 showed no sign of malignancy (controls) (Table IV). Only KCl-Cu was used as "antigen". Each sample was examined independently by two investigators well experienced with the Zeiss cytopherometer. The clinical diagnosis was not known to them. In the 28 patients suffering from a malignant gynaecological tumour, investigator A reported 17 positive and 11 negative test results, the corresponding figures for investigator B being 21 and 7. However, in only 20 of the 28 samples did both investigators report the same qualitative result (15 both positive and 5 both negative), thus precluding a mutual evaluation in 8 cases. Therefore, these 8 samples were re-examined in the cytopherometer by both investigators, giving then 6 positive and 2 negative unanimous test results by both investigators. Thus, 21 tests could be defined finally by both investigators as positive and 7 as negative. In the 20 patients used as controls, 17 cases were classified as test-negative and 3 as positive (included in the 17 are 5 classified as negative only after retesting).

In the third series, from 6 of the 13 patients with an adenocarcinoma of the corpus uteri a double amount of blood could be extracted, enabling duplicate testing (Table V). The samples were arranged in such a way that neither of the investigators knew the diagnosis, nor which samples were duplicates. Independently of each other, the investigators found corresponding results in 6 of the 12 tested samples (Pts A, B, C), 4 of which were classified as positive (Pts A and B), 2 as negative (Pt C) (Table V). The test result of 2 samples (Pts D and E) were positive whereas their duplicates were negative. In one patient (F) both investigators found a negative and in the duplicate a positive result. Re-testing became necessary in 5 cases, since the two investigators obtained discrepant results. Three samples were finally classified as positive, two as negative.

TABLE IV.—*EMT results of the 3rd series using KCl-Cu as "antigen" independently measured by 2 investigators (A and B)*

| Clinical diagnosis | Results of EMT (first testing): + A | + B | − A | − B | Final results of EMT (after retesting): + | − |
|---|---|---|---|---|---|---|
| Ca cervix uteri | 6 | 7 | 3 | 2 | 7 | 2 |
| Ca corpus uteri | 8 | 10 | 5 | 3 | 10 | 3 |
| Ca ovary | 1 | 2 | 3 | 2 | 2 | 2 |
| Ca vulva | 2 | 2 | 0 | 0 | 2 | 0 |
| Total (carcinomas) | 17 | 21 | 11 | 7 | 21 (75%) | 7 (25%) |
| Controls | 4 | 7 | 16 | 13 | 3 (15%) | 17 (85%) |

TABLE V.—*EMT results of duplicate samples of 6 patients (clinical diagnosis: adenocarcinoma corpus uteri) from the 3rd series (2 examiners, A and B)*

| Pt. | Code number: Sample | Code number: Duplicate | First results: A | First results: B | Retesting (on failure of A & B to agree) | Final result |
|---|---|---|---|---|---|---|
| A | 32 | | + | − | yes | + |
| | | 40 | + | + | | + |
| B | 42 | | − | + | yes | + |
| | | 43 | + | − | yes | + |
| C | 6 | | − | − | | − |
| | | 14 | + | − | yes | − |
| D | 16 | | + | + | | + |
| | | 19 | + | − | yes | − |
| E | 52 | | + | + | | + |
| | | 54 | − | − | | − |
| F | 45 | | − | − | | − |
| | | 49 | + | + | | + |

TABLE VI.—*Classification of EMT results according to the clinical stage of the carcinoma*

| Clinical diagnosis | EMT + | EMT − | Concordance EMT/Clin. (%) |
|---|---|---|---|
| Carcinoma *in situ*/ severe dysplasia cervix uteri | 9 | 12 | |
| Ca cervix uteri | 38 | 14 | 73 |
| Stage I | 18 | 4 | 82 |
| II | 10 | 6 | 63 |
| III | 9 | 2 | 82 |
| IV | 1 | 2 | 33 |
| Ca corpus uteri | 39 | 26 | 60 |
| Stage I | 23 | 18 | 56 |
| II | 3 | 5 | 38 |
| III | 11 | 3 | 79 |
| IV | 2 | 0 | 100 |

### DISCUSSION

After the introduction of the MEM test in 1970, a number of independent groups have published reports sometimes with an astonishing amount of agreement between the test results and the clinical diagnosis, thus confirming Field and Caspary's proposal that the test is a valuable tool for cancer diagnosis. Nevertheless, neither the MEM nor its simplified and allegedly equally efficient modifications (MOD–MEM, EMT) gained widespread recognition as a practicable cancer test, and dispute about the quality and value of this test system in providing immunological evidence for malignant tumours is still unresolved. This caused us to carry out our own testing with blood samples from 278 patients using the three "antigenic" proteins, EF, CaBP and KCl-Cu, described in literature.

In the first series, with 150 patients, only EF was used as "antigen". In the second series, CaBP and KCl-Cu were also applied in 80 patients. In the third series 48 patients were tested exclusively with KCl-Cu. After the incubation procedure the samples of the third series were evaluated independently by two investigators and the extent of slowing or acceleration was recorded separately. Without the investigators' knowledge, duplicate samples from 6 patients with adenocarcinoma of the corpus uteri were introduced into the third series.

Although precancerous stages like cis/sD of the cervix cannot be classified as malignant, they are, as a subgroup, listed among the carcinomas in this investigation since MEM as well as EMT results are reported to be predominantly positive (Porzsolt *et al.*, 1975; Klausch *et al.*, 1975; Bögelspacher *et al.*, 1980). According to these authors, this proves the sensitivity of the test, for, they allege, the test was able to detect even the minute changes caused by precancerous diseases. Of the 21 precancerous cases examined by us, 9 showed the positive result expected for malignancy; in 12 the result was negative.

Therefore, at least in our hands, EMT gave no clear-cut answer and was of no diagnostic help.

In examining patients with proven malignant tumours the best rate we could achieve was 75% agreement between the test results and the clinical diagnoses (3rd series). Taken together, the 3 series averaged 63% correlation between EMT and clinical diagnosis. Excluding the cases with cis/sD, the average was 66%. In 37% of the tested patients the result was negative despite the presence of histologically proven malignancy (without the cis/sD cases: 34%).

In the first series, in which 86 patients with confirmed malignancy were tested with EF as "antigen", only 63% positive and 37% negative results were obtained. The worst accuracy was obtained in patients with carcinoma of the corpus uteri; in only 51% did the test correlate with the clinical diagnosis.

In order to improve on these disappointing results, in the second series we tried to apply more specific "antigens". Lymphocytes from 19 of these cancer patients were stimulated in addition to EF with CaBP, and from 12 with KCl-Cu as "antigenic proteins". With 65% positive tests, the results with EF just about equalled those of the first series, whilst the substitution of CaBP as antigen achieved only 58% positive results (Table II).

If we correlate the results of the EF incubation with those of the CaBP incubation, without taking into account the clinical diagnosis, it is striking that in 8 cases (42%) the test result differed according to "antigen", EF or CaBP, was applied. Three times the test with CaBP was positive (correct), but negative (false) in the EF system; 5 times *vice versa* (Table III).

In the 12 samples incubated with KCl-Cu the result was correct (*i.e.* positive) 10 times (83%). However, if we correlate these results with the results of incubation with the other two antigens of samples from the same patients, we receive corresponding results in only 7 cases (58%) compared with EF incubation and in 8 cases (67%) compared with CaBP incubation. Of 10 samples incubated with all 3 antigens, only 6 showed 3 correct results; 4 gave a different result in one system from that with the other two.

The more favourable results obtained with KCl-Cu could only be regarded as tentative owing to the small number of samples tested. Therefore, in the 3rd series only KCl-Cu was used. Also, after incubation the samples were evaluated in the cytopherometer independently by two investigators. In their first evaluation they unanimously declared only 15/28 samples of the carcinoma group as positive (58%). Re-testing of 8 samples, which had at first been analysed differently by the two investigators, produced another 6 positive evaluations: a total of 21 positive cases (Table IV). In this way, 75% of the cases the test results could finally be declared positive, and in accordance with the clinical diagnosis of cancer. However, only 13/28 patients suffered from carcinoma of the corpus uteri. Of these 13, 10 (77%) were correctly identified by both investigators (3 re-testings had been necessary). But a positive EMT result was also obtained in 7/9 patients with squamous carcinoma of the cervix, in 2/4 with a carcinoma of the ovaries and in 2 with a carcinoma of the vulva. These results are surprising, in that according to the publications of Müller *et al.* (1975) application of "organ-specific" KCl-Cu should cause only lymphocytes of patients with an adenocarcinoma of the corpus uteri to react by the production of MSF, *i.e.* the "specific response of lymphokines". According to these authors, lymphocytes from all patients with cancer in a different location or with no cancer at all (controls) ought to show no reaction whatsoever (negative results).

In the 3rd series we also introduced duplicate samples from 6 of the 13 patients with corpus carcinoma without the knowledge of the investigators (Table V). In 3 patients, duplicate samples were analysed by both investigators, but only in 2 of these did the test result correspond to the clinical diagnosis. In one case, both investigators arrived at a false-negative result in the duplicate samples of the same patient. In the remaining 3 patients duplicate samples yielded different results.

At first sight, Table IV might suggest that Investigator B's results more often agreed with the clinical diagnosis than those of Investigator A. However, if we add the results shown in Table V we arrive at a correct quote of 8 for Investigator A compared to only 5 for Investigator B (16 mutually tested samples). Moreover, Investigator B shows a higher rate of false positive values (controls in Table IV); *i.e.* 7 compared to 4 by Investigator A in 20 control tests. Both investigators thus seem to be equally skilled in handling the system.

It is conceivable that in advanced cancer the immunological reactivity of the organism is exhausted and that the respective lymphocytes are therefore not "marked" any more, making them undetectable in the test system, the results of which would remain negative despite the clinical diagnosis of cancer (Field, 1973). It is also possible that with growing numbers of malignant cells (growing tumour mass) an increased number of sensitized mononuclear cells are bound to the tumour itself, thus reducing their number in the periphery and their availability for testing (sponge phenomenon, Field & Caspary, 1972). If one of these theoretical considerations were the cause of the poor correlation between the test results and the clinical diagnosis in our investigation, one would expect the rate of correct test results to vary with the size or stage of the tumour. That is why the results of all 3 series were tabulated for cis/sD and for the stages of the portio and the carcinoma of the body of the uterus in groups according to the clinical stage (Table VI). It shows varying percentages of correct results within the difference groups, but their correlation to the stage of the carcinoma seems random.

TABLE VII.—*EMT results of all 3 test series* (+/−)

| Clinical diagnosis | Series I | Series II | Series III | Total |
|---|---|---|---|---|
| Ca cervix uteri | 20/7 | 11/5 | 7/2 | 38/14 |
| Ca corpus uteri | 21/20 | 8/3 | 10/3 | 39/26 |
| Ca ovary | 12/4 | 2/4 | 2/2 | 16/10 |
| Ca vulva | 1/1 | 3/2 | 2/0 | 6/3 |
| Ca breast | 0/0 | 4/1 | 0/0 | 4/1 |
| Total (carcinomas) | 54/32 | 28/15 | 21/7 | 103/54 |
| Concordance (%) | 63 | 65 | 75 | 66 |
| Carcinoma *in situ*/ severe dysplasia cervix uteri | 8/6 | 1/6 | 0/0 | 9/12 |
| Postmenopausal bleeding | 1/13 | 1/4 | 2/6 | 4/23 |
| Uterusmyoma | 2/8 | 0/2 | 0/2 | 2/12 |
| Incontinence | 2/4 | 1/5 | 0/0 | 3/9 |
| Sterilization | 3/9 | 2/7 | 0/0 | 5/16 |
| Pregnancy | 0/8 | 0/3 | 1/4 | 1/15 |
| Benign ovarian tumours | 0/0 | 2/3 | 0/0 | 2/3 |
| Adnexitis | 0/0 | 0/0 | 0/5 | 0/5 |
| Total (controls) | 8/42 | 6/24 | 3/17 | 17/83 |
| Concordance (%) | 84 | 80 | 85 | 83 |

Negative results were established with the EMT in 83% of the 100 patients who served as controls and were hospitalized for non-malignant diseases. This rate differed between 80 and 85% within the single series (Table VII). Interpretation of the so-called false positive values is difficult—unless we assume complete ineffectiveness of the test system. Although no malignant disease had been detected in these patients, the existence of an occult or micro-carcinoma undetected to this moment cannot be excluded with certainty for every single case; it is conceivable that MEM/EMT tests give accurate positive results at a very early stage in the development of the cancer, long before there is any clinical manifestation of the cancer. However, a 15–20% rate seems too high for this assumption.

A compilation of the individual results of all 3 series is shown in the Figure, in which the percentages of slowing and acceleration are shown graphically. From the second series only the results with EF are considered. An acceptable "discrimination line" to distinguish between malignant (cancer) and non-malignant disease is not recognizable.

FIG.—Individual results of acceleration and inhibition in EMT of the 3 series. Dashed line: arbitrary lower limit of "positives".

### CONCLUSION

Critical and comparative evaluation of the results of the tests with the three "antigens" (Table II), as well as the analyses of the individual results obtained by the two investigators (Table III), suggest poor reproducibility of the EMT procedure in general. The system has at least three weaknesses:

(1) the indicator particles
(2) the so-called "antigens"
(3) the handling of the cytopherometer.

The call for simplification of the indicator cells seemed to have been satisfied with the introduction of the ETS. Whether we are dealing here with the same reactive mechanism for the "cellular immune reaction" as when using macrophages is still to be seen.

From an immunological point of view the "antigenic" preparations are crude extracts containing a number of different proteins. If the test really works on an immunological basis these extracts should be purified. The first question is, however, whether the fundamental part of the test procedure—handling of the cytopherometer—is capable of supplying reproducible and reliable results. The performance of this instrument, as well as the personal skill in handling, seem to be decisive for the test procedure. Since this is a precondition for any improvement—be it isolation of purified and more defined antigens or development of new indicator cells—its value for the MEM, MOD-MEM or EMT, can only be estimated by means of a reliable method of cytopherometry. It was thus our primary task to evaluate the physical possibilities and technical limitations of the Zeiss cytopherometer, which is used by most investigators working with the EMT-MEM or MOD-MEM tests. These results are presented in the following paper.

This study was carried out with financial support from the Bundesministerium für Forschung und Technologie (BMFT), Grant N:01-VH 047-MT-225a and is part of the Habilitationsschrift of one of us (W.H.)

### REFERENCES

ARVILOMMI, H., DALE, M. M., DESAI, H. N., MONGAR, J. L. & RICHARDSON, M. (1977) Failure to obtain positive MEM test in either cell-mediated immune conditions in the guinea pig or in human cancer. *Br. J. Cancer*, **36**, 545.

BAGSHAWE, K. D. (1973) Role of immunity in diagnosis of human cancer. *Br. J. Cancer*, **28** (Suppl. I), 240.

BAGSHAWE, K. D. (1977) Workshop on Macrophage Electrophoretic-mobility (MEM) and Structuredness of Cytoplasmic Matrix (SCM) Tests. *Br. J. Cancer*, **35**, 701.

BAGSHAWE, K. D. (1978) Macrophage electrophoretic mobility and structuredness of cytoplasmic matrix. *Antibiotics Chemother.*, **22**, 155.

BÖGELSPACHER, H. R., TAUTZ, CH. & GEPPERT, M. (1980) Der Elektrophorese-Mobilitäts-(EM-) Test bei präinvasiven dysplastischen Epithelveränderungen an der portio vaginalis uteri. *Geburtshilfe Frauenheilkd.*, **40**, 545.

BØYUM, A. (1968) Separation of leucocytes from blood and bone marrow. *Scand. J. Clin. Lab. Invest.*, **21** (Suppl.), 97.

CARNEGIE, P. R., CASPARY, E. A. & FIELD, E. J. (1973) Isolation of an "antigen" from malignant tumours. *Br. J. Cancer*, **28** (Suppl. I), 219.

CASPARY, E. A. & FIELD, E. J. (1970) Sensitisation of blood lymphocytes to possible antigens in neurological disease. *Eur. Neurol.*, **4**, 257.

CHIU, B., HAUSE, L., ROTHWELL, D., KOETHE, S. & STRAUMFJORD, J. (1977) Effects of encephalitogenic factor on lymphocytic electrophoretic mobility for cancer patients and controls. *Br. J. Cancer*, **36**, 288.

CROZIER, E. H., HOLLINGER, M. E., WOODEND, B. E. & ROBERTSON, J. H. (1976) An assessment of the macrophage electrophoretic mobility test (MEM) in cancer diagnosis. *J. Clin. Pathol.*, **29**, 608.

DICKINSON, J. P., CASPARY, E. A. & FIELD, E. J. (1973) A common tumour specific antigen I. Restriction *in vivo* to malignant neoplastic tissue. *Br. J. Cancer*, **27**, 99.

DOUWES, F. R., SPELLMANN, H. J., MROSS, K. & WOLFRUM, D. I. (1978) Immunodiagnosis of malignant disease VI. Electrophoretic Mobility Test (EMT) in malignant melanoma. *Oncology*, **35**, 163.

DYSON, J. E. D. & CORBETT, P. J. (1978) Effect of lymphocyte supernatants on the electrophoretic mobility of the erythrocytes: Significance in cancer diagnosis. *Br. J. Cancer*, **38**, 401.

EDITORIAL (1976) MEM and MOD-MEM. *Lancet*, i, 897.

FIELD, E. J. (1973) Immunological diagnosis of cancer. In *Modern Trends in Oncology*. Ed. Raven, London: Butterworths, p. 183.

FIELD, E. J. (1976) The immunological diagnosis of human malignant disease. *Ann. Clin. Biochem*, **13**, 495.

FIELD, E. J. & CASPARY, E. A. (1970) Lymphocyte senstitisation: An *in-vitro* test for cancer? *Lancet*, ii, 1337.

FIELD, E. J. & CASPARY, E. A. (1972) Lymphocyte sensitization in advanced malignant disease: A study of serum lymphocyte depressive factor. *Br. J. Cancer*, **26**, 164.

FIELD, E. J., CASPARY, E. A. & SMITH, K. S. (1973) Macrophage electrophoretic mobility (MEM) test in cancer: A critical evaluation. *Br. J. Cancer*, **28** (Suppl. I), 208.

FIELD, E. J. & SHENTON, B. K. (1975) The macrophage electrophoretic mobility test (MEM): A consideration of the practical difficulties and applications of the method. *IRCS Med. Sci.*, **3**, 583.

FORRESTER, J. A., DANDO, P. M., SMITH, W. J. & TURBERVILLE, C. (1977) Failure to confirm the macrophage electrophoretic mobility test in cancer. *Br. J. Cancer*, **36**, 537.

GOLDSTONE, A. H., KERR, L. & IRVINE, W. J. (1973) The macrophage electrophoretic migration test in cancer. *Clin. Exp. Immunol.*, **14**, 469.

GÜNTHER, M., FRIEDRICH, A., ERDMANN, T. & 4 others (1978) A contribution to immunological tumour diagnostics in urology. *Int. Urol. Nephrol.*, **10**, 111.

HARLOS, J. P. & WEISS, L. (1978) Comparison between the macrophage electrophoretic mobility (MEM) and the fixed tanned erythrocyte electrophoretic mobility (FTEEM) tests in the detection of cancer. *Int. J. Cancer*, **21**, 413.

IRMSCHER, J., MÜLLER, M., FISCHER, R., OTTO, G. & STRIEZEL, M. (1975) Makrophagen-Elektrophorese-Mobilitäts-Test (MEM) zur immunologischen Diagnose maligner Geschwülste. *Dtsch. Gesundh.-Wesen*, **30**, 687.

JENSSEN, H. L., KÖHLER, H., GÜNTHER, J. & 4 others (1976*a*) Macrophage-electrophoretic-mobility (MEM)-test in malignant gynecological disease. *Arch. Gynäkol.*, **220**, 191.

JENSSEN, R., JENSSEN, H.-L., KÖHLER, H. & FRIEMEL, H. (1976*b*) The use of the macrophage electrophoretic mobility test for the diagnosis of eye diseases. *Mod. Probl. Ophthal.*, **16**, 259.

JENSSEN, H. L. & SHENTON, B. K. (1975) EMT for lymphocyte sensitization using tanned sheep erythrocytes. *Acta Biol. Med. Ger.*, **34**, 29.

KLAUSCH, B., STRAUBE, W., HOFMANN, R. & 4 others (1975) Erfahrungen mit dem Makrophagen-Elektrophorese-Mobilitäts-Test (MEM-Test) bei der Diagnostik maligner gynäkologischer Erkrankungen. *Zbl. Gynäkol.*, **97**, 529.

KLAUSCH, B., STRAUBE, W., HOFMANN, R. & 4 others (1977) The macrophage electrophoretic mobility (MEM)-test for the diagnosis of hydatidiform mole and chorio carcinoma. *Ann. Chir. Gynaecol.*, **66**, 209.

KREIENBERG, R., SCHÜTZ, G., MELCHERT, F. & LEMMEL, E. M. (1979) Der Elektrophorese-Mobilitats-Hemmtest (EMT) zur immunologischen Frühdiagnostik gynäkologischer Malignome. *Geburtshilfe Frauenheilkd.*, **39**, 709.

LAMPERT, F., NITZSCHKE, U. & ZWERGEL, T. (1977) Lymphocyte sensitization in childhood solid tumours and lymphoblastic leukaemia, measured by electrophoretic mobility test. *Br. J. Cancer*, **35**, 844.

LEADING ARTICLE (1973) Macrophage electrophoretic migration test for cancer. *Nature*, **244**, 130.

LEWKONIA, R. M., KERR, E. J. L. & IRVINE, W. J. (1974) Clinical evaluation of the macrophage electrophoretic mobility test for cancer. *Br. J. Cancer*, **30**, 532.

LIGHT, P. A. & PREECE, A. W. (1977) The use of granulocytes as indicator cells to replace macrophages in the MEM test. *Scand. J. Immunol.*, **6**, 1176

MELTZER, M. S., LEONARD, E. J., RAPP, H. J. & BORSOS, T. (1973) Tumor-specific antigen solubilized by hypertonic potassium chloride. *J. Natl. Cancer Inst.*, **47**, 703.

MEYER-RIENECKER, H., JENSSEN, H. L., KÖHLER, H. & GÜNTHER, J. K. (1975) Zur Bedeutung des Makrophagen-Elektrophorese-Mobilitätstest für die Diagnostik der Geschwülste des Zentralnervensystems. *Dtsch. Med. Wschr.*, **100**, 538.

MOORE, M. & LAJTHA, L. G. (1977) Lymphocyte responses to human tumor antigens: Their role in cancer diagnosis. In *International Review of Experimental Pathology*, Ed. Richter & Epstein. London and New York: Academic Press. p. 97.

MOORE, M. (1978) Human tumour-associated antigens: Methods of *in vitro* detection. In *Immunological Aspects of Cancer*, Ed. Castro. Lancaster: MTP Press. p. 52.

MÜLLER, M., IRMSCHER, J., FISCHER, R. & GROSSMANN, H. (1975) Immunologisches Tumorprofil: Ein neuartiges Prinzip in der Anwendung des Makrophagen-Elektrophorese-Mobilitäts (MEM)—Test zur differenzierten Karzinomdiagnose. *Gesundh. Wesen*, **30**, 1836.

MÜLLER, M., IRMSCHER, J., FISCHER, R., HEIDL, G. & GROSSMANN, H. (1977) Immunological tumour profile: Organ-specific carcinoma diagnosis in patients employing the macrophage electrophoretic mobility test. *Cancer Lett.*, **2**, 139.

NAKAJIMA, T., CHIKAMORI, M., ISOJIMA, K. & IWAGUCHI, T. (1977) Lymphocyte reactivity to allogenic tumor antigens and myelin basic protein in gastric cancer patients. *Gann*, **68**, 449.

NOWAK, R., JENSSEN, H. L., KÖHLER, H., WERNER, H., KRAMP, B. & PUTZKE, H.-P. (1976) Zur Anwendung des Makrophagen-Elektrophorese-Mobilitäts-Testes in der Diagnostik maligner Tumoren der Hals-Nasen-Ohren-Heilkunde. *HNO-Praxis*, **2**, 94.

PORZSOLT, F., MÜHLBERGER, G. & AX, W. (1975) Electrophoretic mobility test (EMT): II. Is there a correlation between the clinical diagnosis and immunologic test for precancerous diseases? *Behring Inst. Mitt.*, **57**, 137.

PORZSOLT, F., TAUTZ, CH. & AX, W. (1975) Electrophoretic mobility test: I. Modifications to simplify the detection of malignant diseases in man. *Behring Inst. Mitt.*, **57**, 128.

PREECE, A. W. & LIGHT, P. A. (1974) The macrophage electrophoretic mobility (MEM) test for malignant disease. Further clinical investigations and studies of macrophage slowing factors. *Clin. Exp. Immunol.*, **18**, 543.

PRITCHARD, J. A. V., MOORE, J. L., SUTHERLAND, W. H. & JOSLIN, C. A. F. (1972) Macrophage-electrophoretic-mobility (MEM)—test for malignant disease: An independent confirmation. *Lancet*, i, 627.

PRITCHARD, J. A. V., MOORE, J. L., SUTHERLAND, W. H. & JOSLIN, C. A. F. (1973) Evaluation and development of the macrophage electrophoretic mobility (MEM) test for malignant disease. *Br. J. Cancer*, **27**, 1.

PRITCHARD, J. A. V., MOORE, J. L., SUTHERLAND, W. H. & JOSLIN, C. A. F. (1976) Clinical assessment of the MOD-MEM cancer test in controls with non-malignant diseases. *Br. J. Cancer*, **43**, 1.

PRITCHARD, J. A. V., SUTHERLAND, W. H., TRESDALE, C., WHITEHEAD, R. H., DEELY, T. J. & HUGHES, L. E. (1978) The MEM-test—an investigation of its value as a routine laboratory test in the detection of malignant disease. *Ann. Clin. Res.*, **10**, 71.

RAHI, A. H. S., OTIKO, G. & WINDER, A. F. (1976) Evaluation of macrophage electrophoretic mobility (MEM) test as an indicator of cellular immunity in ocular tumours. *Br. J. Ophthalmol.*, **60**, 589.

RAWLINS, G. A., WOOD, J. F. M. & BAGSHAWE, K. D. (1976) Macrophage electrophoretic mobility (MEM) with myelin basic protein. *Br. J. Cancer*, **34**, 613.

RITTER, J. & OEHME, J. (1978) Erfahrungen mit dem Elektrophorese Test bei Kindern. *Mschr. Kinderh.*, **126**, 556.

SHENTON, B. K., JENSSEN, H. L., WERNER, H. & FIELD, E. J. (1977) A comparison of the kinetics of the macrophage electrophoretic mobility (MEM) and the tanned sheep erythrocyte electrophoretic mobility (TEEM) tests. *J. Immunol. Meth.*, **14**, 123.

TAUTZ, CH., SCHNEIDER, W., LAIER, E. & BRÜGMANN, G. (1978) Der Elektrophorese-Mobilitäts-(EM) Test: Untersuchungsmethode zur unterscheidung von malignen und nicht malignen Tumoren. *Klin. Wschr.*, **56**, 175.